# AKR7A5 knockout promote acute liver injury by inducing inflammatory response, oxidative stress and apoptosis in mice

**DOI:** 10.1111/jcmm.70129

**Published:** 2024-10-04

**Authors:** Hui Shi, Wenda Xu, Qingling Liu, Yan Li, Silin Dong, Zhenjun Zhao

**Affiliations:** ^1^ College of Life Science Yantai University Yantai China

**Keywords:** acute alcohol injury, AKR7A5, apoptosis, coagulation and haemostasis, oxidative stress

## Abstract

Alcohol liver disease has become a worldwide critical health problem. The ingested alcohol could be converted into acetaldehyde or combined with free fatty acids to induce the endoplasmic reticulum oxidative stress in the liver. Coincidentally, AKR7A5 has both aldehyde detoxification and antioxidant effects. Therefore, we discuss the possible role and mechanism of AKR7A5 in the acute alcohol injury of mice liver. There were four experiment groups, the C57BL/6 mice of wild‐type mice (WT) or AKR7A5−/− mice (KO) were intragastrically administrated with saline or 50% ethanol at 14 mL/kg, respectively. Compared to the WT + alcohol group, abnormal liver function, disordered hepatic cord, severe congestion in the hepatic sinus and the space of the hepatic cord, occurrence of oxidative stress, DNA damage and different expressions of apoptosis‐related proteins were detected in the KO + alcohol group. Meanwhile, the biological process enrichment analysis showed that the down‐regulated proteins were related to the metabolism of fatty acid, the up‐regulated proteins positive regulation of reactive oxygen species metabolic process, negative regulation of coagulation and haemostasis. In conclusion, single ethanol binge combined with the absence of AKR7A5 caused more severe inflammatory response, oxidative stress, apoptosis of endogenous pathways, abnormal lipids metabolism and disordered coagulation in mice liver.

## 1 INTRODUCTION

Alcohol liver disease (ALD) has become a critical health problem all over the world, which is the second major cause of liver injury following viral hepatitis.^1^ ALD is related to a range of disorders, including simple steatosis, steatohepatitis, cirrhosis and end‐stage hepatocellular carcinoma (HCC).^2^ Although the mechanisms and pathology of ALD have been gained many knowledge, the research results are great variety, which may be due to the variety of animal models for ALD. In the last decades, numerous rodent models of chronic or acute alcohol exposure have been established and extensive evidences have been accumulated on the pathological process of ALD (Table 1).^2^ In addition, some models of gene‐deficient mice, such as human CYP2E1 and p47phox NADPH oxidase, also have been established.^3^ However, the recapitulation of the full spectrum of ALD is limited, and many ALD‐associated genes of interest have not been fully identified.

**TABLE 1:** Rodent models of alcoholic liver disease.

References	Treatment	Elevation of ALT	Liver fibrosis	Steatosis	High blood alcohol levels	Inflammation	Elevated acetaldehyde levels	Lipid peroxidation	Necrosis
**[** 28, 29]	Chronic ethanol feeding (4–12 week)	**√**	**×**						
	Chronic ethanol feeding + single/multiple binges (4–6 week)	**√**	**×**	**√**					
	Oral alcohol in drinking water (10 d/1–2 week)	**√**	**×**	**√**					
	Intragastric infusion (2–3 month)	**√**	**×**	**√**					
**[** 30]	LDE + single ethanol binge			**√**	**√**	**√**			
	LDE + 3 ethanol binges				**√**				
**[** 31]	Ethanol + CCl4 treatment		**√**				**√**		
**[** 16]	6 g/kg alcohol given by gavage	**√**		**√**				**√**	**√**

Abbreviations: ×, no symptom; blank, no mention; √, present symptoms; CCl_4_, Carbon tetrachloride; LDE, Lieber‐De Carli ethanol diet.

The Aldo‐keto reductase (AKR) superfamily is a significant metabolic enzyme of aldehyde detoxification,^3^, ^4^ which can reduce the aldehydes to their corresponding alcohols. The AKR7A subfamily has received particular attention, due to the valuable therapeutic approaches for diseases related to reactive carbonyls.^5^ So far, five members of ARK7A have been identified: two human members‐AKR7A2 and AKR7A3, two rat members‐AKR7A1 and AKR7A4 and one mouse member‐AKR7A5.^6^ AKR7A5 is the only AKR7A enzyme in mice, which has 89% identity to human AKR7A2.^7^ AKR7A5 expresses highly in liver, kidney, testes, brain and heart.^7^ AKR7A5 is enzymatically active against hexanal, trans‐2‐nonenal, methyglyoxal and 4‐hydroxynonenal,^8^ which are the harmful intermediate metabolites of ethanol in the liver.^9^, ^10^, ^11^, ^12^ In addition, the AKR7A5 enzyme can protect against 4‐hydroxynonenal‐induced apoptosis via caspase‐3 activation.^13^ The role of AKR7A5 in resistance to oxidative stress in Chinese hamster lung fibroblasts (V79‐4 cells) has also been verified.^14^

In the liver, the metabolic process of alcohol contains oxidative pathway, by which the ingested alcohol is converted into acetaldehyde (ACE),^15^ and nonoxidative pathway, in which the combination of alcohol with free fatty acids induces the endoplasmic reticulum oxidative stress (Huang et al. 2104). Coincidentally, AKR7A5 has both aldehyde detoxification^8^ and antioxidant effects.^14^ In the present study, we examined whether and how AKR7A5 could protect the liver from acute alcohol injury.

## 2 MATERIALS AND METHODS

### 2.1 Animal model

The C57BL/6 mice of AKR7A5−/− were kindly presented by Shandong University. The C57BL/6 mice of wild type were used as control. The male mice (4 weeks of age, 12–15 g) were used for all experiments. The mice were fed for 7 days to acclimatize to the environment, and the method of ALD modelling referred to Zhou et al.^16^ The mice were intragastric administration with saline of 14 mL/kg for wild‐type mice (WT) of control group (*n* = 10) and AKR7A5−/− mice (KO) of control group (*n* = 10). And the mice were intragastric administration with 14 mL/kg of 50% ethanol for WT alcohol treatment group (*n* = 10) and KO alcohol treatment group (*n* = 10). Before the treatment, all mice were fasted without water for 12 h, then were given saline or ethanol, respectively. After 8 h, the body weight of mice was monitored. The blood sample was collected from the eye socket. Mice were killed by cervical dislocation, and the liver was quickly separated and took photograph under stereomicroscope. Then the organs were stored at −80°C or in Bouin's fixative solution. The study was approved by the Ethics Committee of Yantai University, Yantai, China, on March 13 2021 (3/13/2021, No. YTU‐2021007) and was performed in accordance with the Helsinki declaration.

### 2.2 Histopathological analysis

The samples were fixed in Bouin's fixative solution for 48 h and embedded in paraffin. Standard sections of 4 μm thickness were stained with haematoxylin and eosin (H&E) kit (C0105S, Beyotime, Shanghai, China) according to the instruction manuals, and observed using light microscopy for histological observations. The pathological damage of the liver was quantified as bleeding area (excluded the bleeding areas from hepatic sinus)/total area. The Image Pro Plus software (V6.0) was used to analyse the bleeding area.

### 2.3 Biochemical indicators detection

Under aseptic conditions, the blood sample was collected from the eye socket and placed at room temperature for 30 min, and centrifuged at 3500 rpm for 20 min to obtain the serum samples. The levels of Aspartate aminotransferase (GOT), Alanine aminotransferase (GPT) and Alkaline phosphatase (AKP) in serum were detected using the commercial kits (C010‐2‐1, C009‐2‐1, A059‐2‐2) of Nanjing Jiancheng Bioengineering Institute (Nanjing, China) according to the instruction manuals.

### 2.4 Protein spectrum analysis

The protein spectrum analysis of liver samples were carried out by Beijing Zhengda Kangjian Biomedical Technology Co., LTD using the Orbitrap Fusion Lumos mass spectrometer (Thermo Fisher Scientific, USA). The main experimental procedures were as follows: the liver tissue was digested with trypsin, re‐suspended in buffer A (1.9% acetonitrile and 0.1% formic acid), and then centrifuged at 16000 g for 10 min. The supernatant was further loaded onto a reverse‐phase analytical column (150 μM diameter; ReproSil‐Pur C18‐AQ 1.9 μM resin; pore size 120 Å) and separated using an EASY‐nLC 1200 system (Thermo Fisher Scientific). The eluting peptides were classified and quantified using a Thermo Orbitrap HF mass spectrometer (Thermo Fisher Scientific). The proteins were identified using the Proteome Discoverer search engine. The raw MS files were searched using the MASCOT search engine (version 2.1.04). The support of ≥1 unique peptide was defined as valid proteins. The ratio of the mean values of all biological measurements of each protein in the two compared sample groups (WT + alcohol; KO + alcohol) was taken as the fold change (Fold Change, FC). The median of the protein quantification value was used as the screening condition to identify the statistically significant differential expression protein (DEP). The functions of DEPs were assessed with Gene Ontology (GO) and Kyoto Encyclopedia of Genes and Genomes (KEGG) enrichment analyses. The purpose was to detect whether the DEPs had a significant enrichment trend in some functional types, with *p* value <0.05 as the threshold and the entries that met this condition were defined as significantly enriched entries.

### 2.5 Bioinformatics analysis

The org.Hs.eg.db package (Rstudio based on R3.4.1, Boston, MA) was used for the annotation of identified proteins and conversion of gene ID. DEPs were categorized by k‐means and hierarchical clustering (cluster package, Rstudio based on R3.4.1, Boston, MA). The optimal cluster number was determined and visualized using gap statistics and the pheatmap package (Rstudio based on R3.4.1, Boston, MA), respectively. The Retrieval of Interacting Genes/Proteins (STRING, http://www.string‐db.org/) was used to construct the protein–protein interaction (PPI) network. Interaction modules and hub proteins were analysed with the MCODE and CentiScaPe plugins in Cytoscape 3.5.1 with default parameters. Modules with a MCODE score greater than 4.3692 were selected for further analysis. Proteins with a centrality score greater than the threshold value of 2 were considered as hub‐proteins.

### 2.6 Oxidative stress analysis

A portion of the liver tissues (0.2–1 g) were homogenized with 9 times the volume of physiological saline on ice and centrifuged at 5000 rpm for 15 min. The activities of glutathione peroxidase (GSH), superoxide dismutase (SOD) and total antioxidant capacity (TAC) in the liver were detected using the commercial kits (S0056, S0101S, S0116, Beyotime Biotechnology, Shanghai, China) according to the instruction manuals. The levels of malonaldehyde (MDA) and 4‐Hydroxynonenal (4‐HNE) in the liver were detected using the commercial kits (S0131S, H268‐1‐1, Nanjing Jiancheng Bioengineering Institute, Nanjing, China) according to the instruction manuals.

### 2.7 Apoptosis Assay

The apoptosis assay was conducted on the standard sections of 4 μm thickness. The apoptotic cells were detected using the One Step TUNEL Apoptosis Assay Kit (C1088, Beyotime Biotechnology, Shanghai, China) according to the instruction manuals. The apoptotic cells were labelled with green fluorescence. The proportion of apoptotic cell (apoptotic cells/total cells) was analysed using the Image J software (V1.8.0.112).

### 2.8 Western blot

The total proteins of liver were extracted using RIPA lysis buffer (P0013B, Beyotime Biotechnology, Shanghai, China) supplemented with PMSF (ST506, Beyotime, China). The total proteins were then separated on a 10%–12% (w/v) sodium dodecyl sulphate–polyacrylamide gel. After electrophoresis, transfer membrane and blocking with 5% BSA, the membrane was incubated with primary antibody (Abcam, UK) overnight at 4°C. The dilution ratio of primary antibody used was as follows: Anti‐GAPDH antibody (ab9485) was 1:2000, Anti‐p53 antibody (ab183544) was 1:1000, Anti‐Bcl2 antibody (ab16904) was1:400, Anti‐Bax antibody (ab32503) was 1:1000, Anti‐Caspase9 antibody (ab202068) was 1:2000 and Anti‐Caspase‐3 antibody (ab184787) was 1:2000. After incubation with the primary antibody and washing with TBS‐Tween (1000:1), the membrane was incubated with the relevant HRP‐labelled secondary antibody (Beijing Zhongshan Jinqiao Biotechnology Co. LTD, China) for 2 h at room temperature. The target proteins were detected using the ChemiDoc Imaging System (Bio‐Rad, Hercules, CA, USA). The intensity of targeted proteins was determined using the Image J software (V1.8.0.112).

### 2.9 Statistical analysis

For the statistical analyses between different sample groups, one‐way analysis of variance (ANOVA) of the SPSS 20.0 software was used and the data were presented as means ± standard deviation (SD). *p* values <0.05 was considered statistically significant.

## 3 RESULTS

### 3.1 The alcohol ingestion impaired liver tissue

**FIGURE 1:**
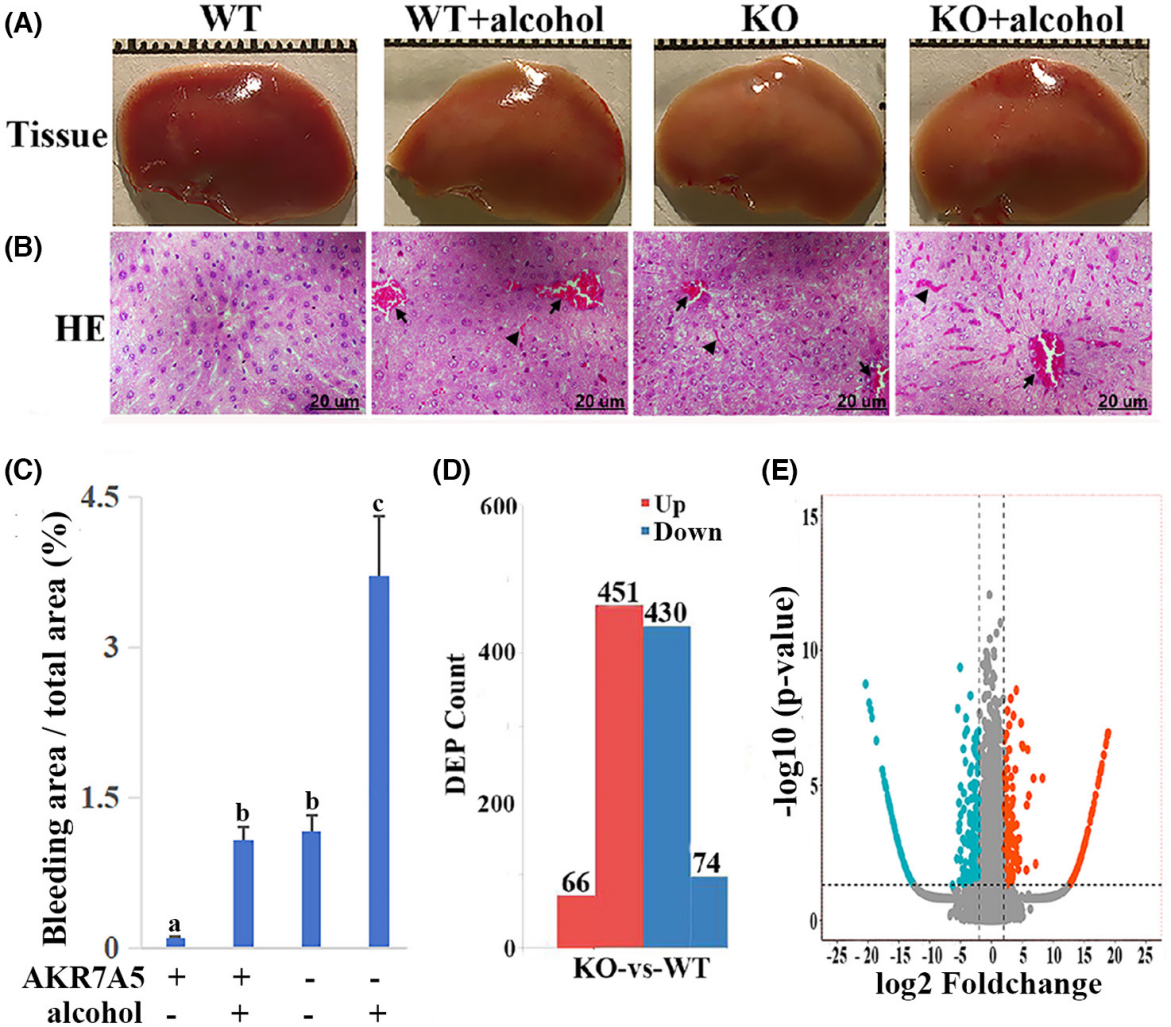
The appearance (A), the pathological change of mice liver (B), the histogram of percentage of bleeding area (C), the histogram of the number of DEPs (D) and the volcano plot of quantifiable proteins identified by LC–MS/MS (E). The liver of the WT control group presented jujube red, and the livers of the other three groups (WT + alcohol, KO control, KO + alcohol) showed different degrees of earthy yellow. The slight bleeding at the liver margin was presented in both WT + alcohol and KO + alcohol groups. Alcohol treatment induced congestion in the hepatic sinus (arrows) and the space of the hepatic cord (arrowheads). Bar = 20 μm. The calculation of bleeding area didn't include bleeding areas of hepatic sinus. The different letter indicated a statistically significant difference between the two groups. Compared to the WT + alcohol group, the number of up‐regulated proteins was 517, in which 66 specific expression proteins in the KO + alcohol group; the number of down‐regulated proteins was 504, in which 74 proteins were not detected in KO + alcohol group. The red spots on the top‐right quadrant indicated significantly up‐regulated proteins (*p* < 0.05), the blue spots on the top‐left quadrant indicated significantly down‐regulated proteins (*p* < 0.05).

The liver of WT control group presented jujube red. The livers of the other three groups (WT + alcohol, KO control, KO + alcohol) showed different degrees of earthy yellow. Compared to the WT control group, the livers of the KO control and KO + alcohol groups were brittle, and there was slight bleeding at the liver margin in both WT + alcohol and KO + alcohol groups (Figure 1A).

In the WT control group, the hepatocyte were orderly arranged and the nucleus were rounded. Compared to the WT group, the WT + alcohol group showed disordered hepatic cords and congestion in the hepatic sinus. The arrangement of hepatic cord is relatively regular, but there were congestion in the hepatic sinus and the space of hepatic cord in the KO control group. Compared to the other three groups, the KO + alcohol group showed more severe congestion in the hepatic sinus and the space of the hepatic cord, and disordered hepatic cords (Figure 1B). The bleeding area of KO + alcohol group was significantly increased compared to the other three groups (*p* < 0.05) (Figure 1C).

The biochemical indicators of liver function were shown in the Table 2. Compared to WT control group, the AKP, GOT and GPT in WT + alcohol group were significantly increased (*p < 0.01). Compared to KO control group, the GOT and GPT in KO + alcohol group were significantly increased (^※^ *p* < 0.01). Compared to WT control group, the AKP and GPT in KO control group were significantly increased (^#^ *p* < 0.01). Compared to WT + alcohol group, the GOT and GPT in KO + alcohol group were significantly increased (^†^ *p* < 0.01).

**TABLE 2:** The levels of AKP, GOT, and GPT in serum of mice.

	Group			
	WT control	WT + alcohol	KO control	KO + alcohol
AKP(U/L)	34.57 ± 0.51	38.53 ± 0.80*	38.67 ± 0.54^#^	39.43 ± 1.06
GOT (U/L)	16.97 ± 1.34	47.04 ± 3.42*	18.80 ± 0.67	96.72 ± 4.43^†^, ^※^
GPT(U/L)	485.92 ± 12.90	703.43 ± 24.95*	598.66 ± 28.15	799.07 ± 42.41^†^, ^※^

*Note*: All data are presented as mean ± SD (*n* = 10).

Abbreviations: AKP, Alkaline phosphatase; GOT, Aspartate aminotransferase; GPT, Alanine aminotransferase.

^#^ KO control group versus WT control group (*p* < 0.01).

^†^ KO + alcohol group versus WT + alcohol group (*p* < 0.01).

* WT + alcohol group versus WT control group (*p* < 0.01).

^※^ KO + alcohol group versus KO control group (*p* < 0.01).

### 3.2 Proteomics analysis

There were 517 up‐regulated proteins and 504 down‐regulated proteins in the KO + alcohol group compared to the WT + alcohol group. Among these DEPs, there were 66 specific expression proteins in the KO + alcohol group, and 74 specific expression proteins in the WT + alcohol group (Figure 1D,E).

**FIGURE 2:**
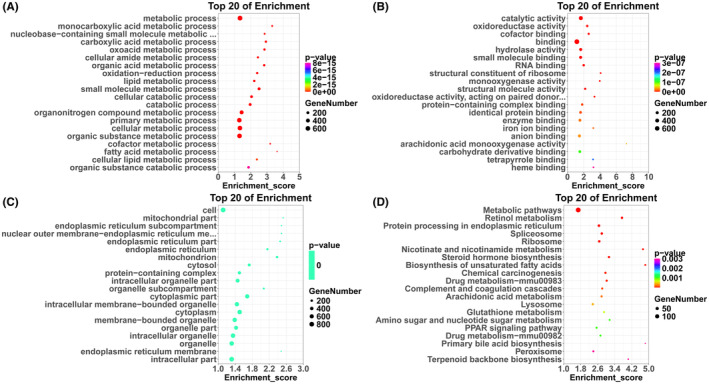
Statistical diagram of Gene ontology (GO) enrichment analysis (A: Biological Process; B: Molecular Function; C: Cellular Component) and Kyoto Encyclopedia of Genes and Genomes (KEGG) pathway enrichment analysis (D) of DEPs in KO + alcohol group versus WT + alcohol group. The colour represented the *p* value. The redder colour represented the more significant enrichment. The size of the dot represented the number of differentially expressed proteins. The Enrichment score refers to the enrichment fold. The higher score indicated the obvious enrichment.

Enrichment analysis revealed that the DEPs obtained principally participated in metabolic and catabolic processes as well as oxidation–reduction processes (BP) (Figure 2A). These effects were exerted through influencing enzymatic activity involving catalytic‐, oxidoreductase‐, hydrolase‐, monooxygenase‐, structural molecule‐, oxidoreductase‐, arachidonic acid monooxygenase‐ and molecular binding involving cofactor‐, small molecule‐, RNA‐, protein‐containing complex‐, identical protein‐, enzyme‐, iron ion‐, anion‐, carbohydrate derivative‐, tetrapyrrole‐, heme‐ (MF) (Figure 2B), mainly within the mitochondria, endoplasmic reticulum, nucleus and cytoplasmic (CC) (Figure 2C).

The KEGG enrichment analysis was then performed on DEPs. Similarly, the DEPs mainly participated in metabolism pathways, such as retinol‐, nicotinate and nicotinamide‐, drug‐, arachidonic acid‐, glutathione‐, amino sugar and nucleotide sugar; biosynthesis pathways, such as steroid hormone‐, unsaturated fatty acids, primary bile acid‐, terpenoid backbone‐, chemical carcinogenesis and PPAR signalling pathway (Figure 2D).

### 3.3 Clustering combined with biological process enrichment analysis revealed up‐regulation of immune response and down‐regulation of metabolic process in the liver of AKR7A5−/− mice compared to the WT under acute alcohol

**FIGURE 3:**
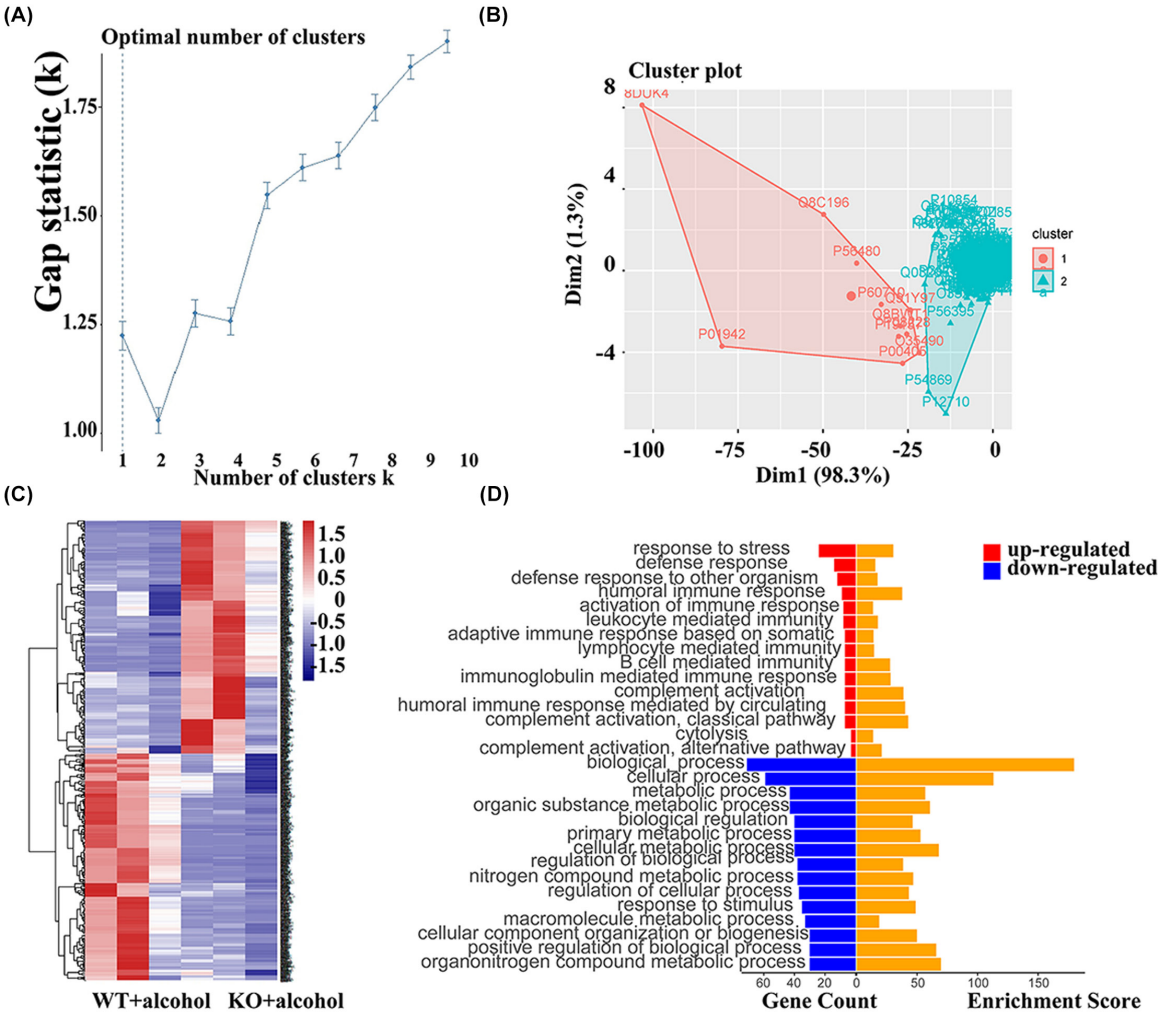
The optimal cluster number was evaluated with gap statistics (A). The optimal *k* value was 2. The K‐means clustering of DEPs was analysed with an optimal *k* value (B). The 2 distinct clusters were revealed, Cluster 1 and Cluster 2. Hierarchical clustering analysis of DEPs applying the optimal *k* value (C). Gene symbols of DEPs were illustrated on the right side. Biological process terms of the top 15 proteins counted in the GO‐BP enrichment analysis for Cluster 1 (bottom 15 bars on the left) and Cluster 2 (top 15 bars on the left) (D).

To further evaluate the functional changes in the liver tissue of AKR7A5−/− mice in response to acute alcohol, K‐means cluster analysis was performed using the factoextra component (Rstudio based on R3.4.1) applied to 140 DEPs (66 specific expression proteins in the KO + alcohol group, and 74 specific expression proteins in the WT + alcohol group). When calculated using the gap statistical method, the best *k* value indicated that the best clustering result was 2 clusters (Figure 3A), which were then visualized as shown in Figure 3B. Hierarchical cluster analysis was then used to evaluate the 140 DEPs, as in R3.4.1. By applying an optimal *k* value of 2, hierarchical clustering produced the same result as clustering with K‐means (Figure 3C). Combining these two clustering methods, 140 DEPs could be divided into two clusters, cluster 1 containing 74 down‐regulated proteins and cluster 2 containing 66 up‐regulated proteins in KO + alcohol group versus WT + alcohol group. To get a clearer picture of these clusters of proteins, a Biological Process (BP) enrichment analysis from GO was used to analyse the two clusters individually. The results of this analysis showed that the down‐regulated proteins of cluster 1 were more enriched in processes involved in the metabolic process and biological regulation, while the up‐regulated proteins of cluster 2 were involved in responses to stress, defence, and activation of immune response, and inflammatory response (Figure 3D).

### 3.4 Protein–protein interaction analysis for the identification of interaction modules and hub proteins of DEPs in the liver of AKR7A5−/− mice compared to the WT under acute alcohol

**FIGURE 4:**
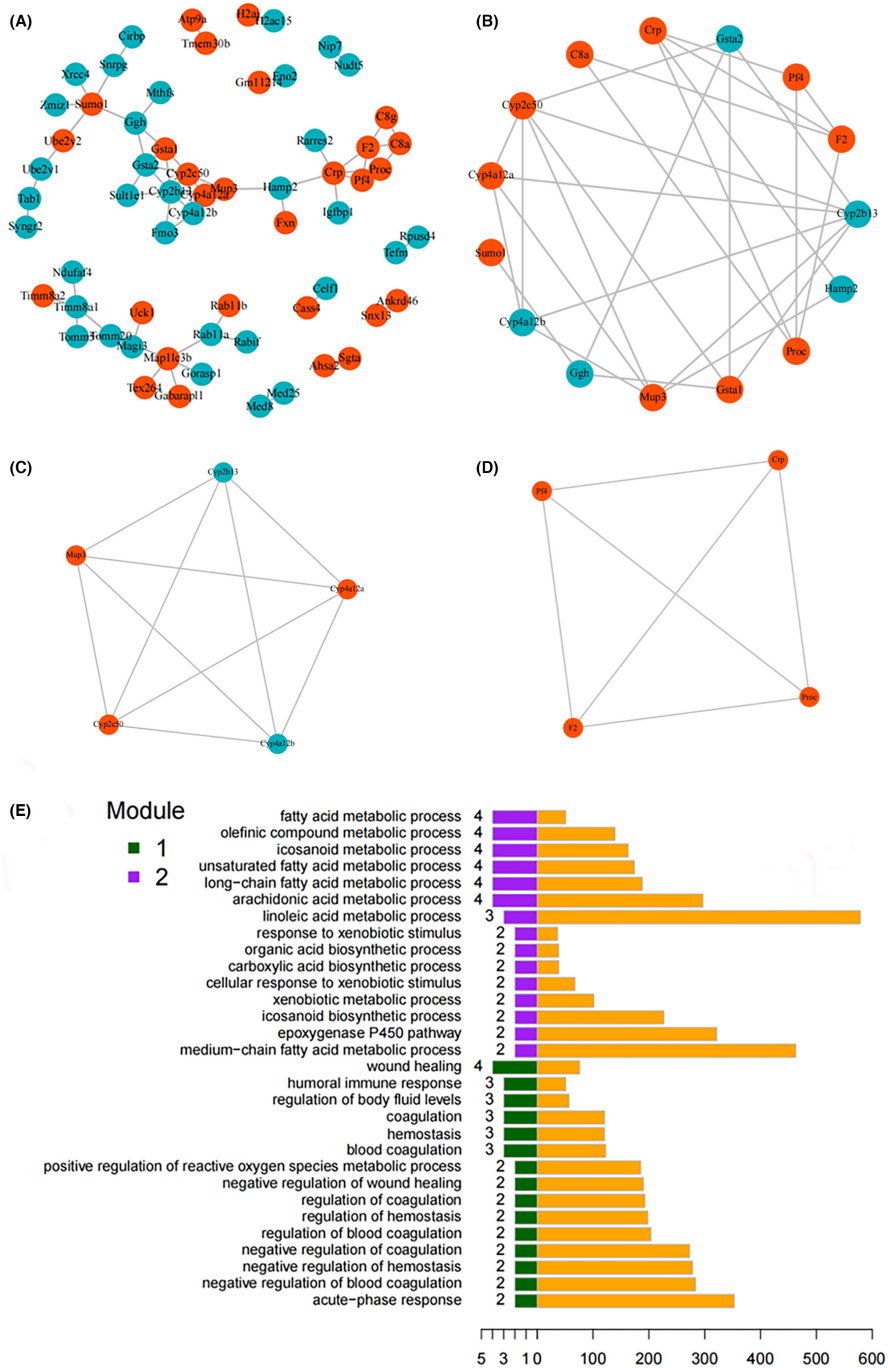
The PPI network of DEPs (A). The red spots indicated up‐regulated proteins, and the blue spots indicated down‐regulated proteins in KO + alcohol group versus WT + alcohol group. The hub proteins identified by CentiScaPe in the PPI network (B). Interaction module 1 (C) and module 2 (D) mined from the PPI network using MCODE analysis. Biological process terms of the top 15 proteins count in the GO‐BP enrichment analysis for Module 1 (bottom 15 bars on the left) and Module 2 (top 15 bars on the left) (E). Bars on the right represented respective enrichment scores.

To explore possible interactions between the 140 DEPs, protein interaction (PPI) scores were calculated using the string online database. After establishing a threshold interaction score of 0.4 (default), a PPI network containing a total of 123 nodes and 52 interactions was identified (visualized in Cytoscape 3.5.1 software) (Figure 4A). The CentiScaPe 15 proteins (*ten up‐regulated*: Prothrombin [Proc], Glutathione S‐transferase alpha [Gstal], Major urinary protein 3 [Mup3], Small ubiquitin‐related modifier 1 [Sumo1], Cytochrome P450 4A12A [Cyp4a12a], Cytochrome P450 2C50 [Cyp2c50], Complement component 8 alpha chain [C8a], C‐reactive protein [Crp], Platelet factor 4 [Pf4], Coagulation factor II [prothrombin] [F2]; and *five down‐regulated*: Cytochrome P450 2B13 [Cyp2b13], Hepcidin antimicrobial peptide 2 [Hamp2], Gamma‐glutamyl hydrolase [Ggh], Cytochrome P450 4A12B [Cyp4a12b], Glutathione S‐transferase alpha‐2 [Gsta2]) to the centre were more significant than the threshold value of 3 degrees from the network filter (Figure 4B). Therefore, these were considered as the hub proteins. Within the entire PPI network, two interaction modules were mined using the MCODE plugin in Cytoscape by setting the cutoff degree to 2 (Figure 4C,D). The BP enrichment analysis of the two modules showed that module 1 was related to the metabolism of fatty acid, unsaturated fatty acid metabolism, long‐chain fatty acid metabolism, arachidonic acid metabolism, olefinic compound, icosanoid. The epoxygenase P450 pathway was also enriched in processes. Meanwhile, module 2 was more enhanced in positive regulation of reactive oxygen species metabolic process, negative regulation of coagulation and haemostasis (Figure 4E).

### 3.5 The alcohol ingestion caused oxidative stress in liver

Compared to WT control group, the SOD activity in WT + alcohol group was significantly decreased (*p < 0.01). Compared to KO control group, the GSH activity, SOD activity and the total antioxidant capacity in KO + alcohol group were significantly decreased; the MDA level in KO + alcohol group was significantly increased (^※^ *p* < 0.01). Compared to WT control group, the SOD activity and total antioxidant capacity in KO control group were significantly decreased; the MDA level in KO control group was significantly increased (^#^ *p* < 0.01). Compared to WT + alcohol group, the GSH activity, SOD activity and total antioxidant capacity in KO + alcohol group were significantly decreased; the MDA level in KO + alcohol group was significantly increased (^†^ *p* < 0.01). There was no significant difference of 4‐HNE level among all groups (Table 3).

**TABLE 3:** The detection index of oxidative stress in the liver of mice.

	Group			
	WT control	WT + alcohol	KO control	KO + alcohol
GSH (U/mgprot)	15.33 ± 3.90	17.48 ± 1.71	17.13 ± 2.18	9.19 ± 0.73^†^, ^※^
SOD (U/mgprot)	4.76 ± 0.13	3.96 ± 0.32*	3.21 ± 0.74^#^	1.43 ± 0.14^†^, ^※^
TAC (μM)	27.31 ± 2.55	25.97 ± 1.68	20.53 ± 1.59^#^	17.98 ± 1.91^†^, ^※^
MDA (μmol/mgprot)	722.40 ± 8.23	720.31 ± 22.61	793.2 ± 14.24^#^	1012.9 ± 20.28^†^, ^※^
4‐HNE (ng/L)	2.25 ± 0.11	2.34 ± 0.01	2.32 ± 0.21	2.28 ± 0.03

*Note*: All data are presented as mean ± SD (*n* = 10).

Abbreviations: 4‐HNE, 4‐Hydroxynonenal; GSH, glutathione peroxidase; MDA, malonaldehyde; SOD, superoxide dismutase; TAC, total antioxidant capacity.

^#^ KO control group versus WT control group (*p* < 0.01).

^†^ KO + alcohol group versus WT + alcohol group (*p* < 0.01).

* WT + alcohol group versus WT control group (*p* < 0.01).

^※^ KO + alcohol group versus KO control group (*p* < 0.01).

### 3.6 The alcohol ingestion caused hepatocyte apoptosis

**FIGURE 5:**
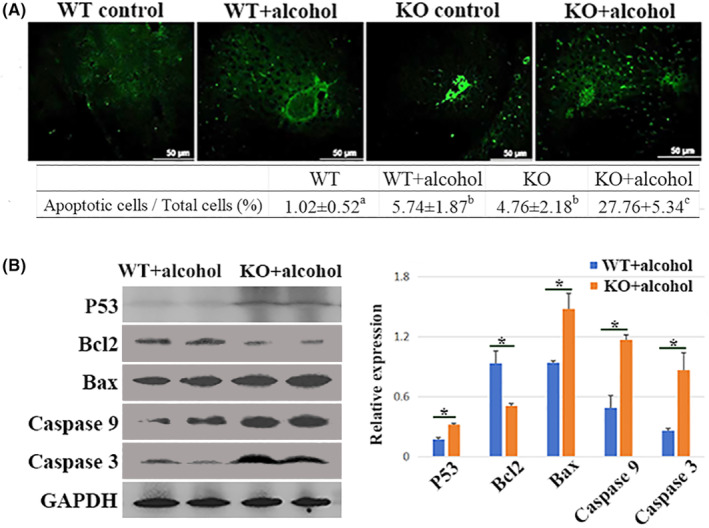
The detection of apoptotic cells with TUNEL assay (A), and the detection of apoptosis‐related proteins by Western blot method (B). Compared to WT control group, the number of apoptotic cell in the WT + alcohol group was increased (*p* < 0.05). The apoptotic cells were significantly increased in the KO + alcohol group compared to the other groups (*p* < 0.01). Compared to WT + alcohol group, the expressions of P53, Bax, Caspase 9 and Caspase 3 were significantly increased in the KO + alcohol group (*p* < 0.05), and the expression of Bcl2 in KO + alcohol group was significantly decreased in the KO + alcohol group (*p* < 0.05). * means *P < 0.05.*

There were occasionally observed apoptotic cells in both WT control and KO control groups. Compared to WT control group, the number of apoptotic cell in the WT + alcohol group was significantly increased (*p* < 0.05). The apoptotic cells were significantly increased in the KO + alcohol group compared to the other groups (*p* < 0.01) (Figure 5A).

The expression of apoptosis‐related proteins were shown in Figure 5B. Compared to WT + alcohol group, the expressions of P53, Bax, Caspase 9 and Caspase 3 were significantly increase in the KO + alcohol group (*p* < 0.05). Meanwhile, the expression of Bcl2 in KO + alcohol group was significantly decreased (*p* < 0.05).

## 4 DISCUSSION

Following absorption in the gastrointestinal tract (GI), only 2%–10% of total ethanol absorbed by the GI is directly eliminated through the lung, kidney and sweat in an unchanged form,^17^ and most ethanol undergo the metabolic processing in the liver.^2^ In the liver, the metabolic process of single ingested alcohol contains oxidative and non‐oxidative pathways. In the oxidative pathway, ingested alcohol is converted into ACE, subsequently ACE is metabolized into acetate.^18^ In the non‐oxidative pathway, alcohol combined with free fatty acids and caused organ inflammation and injury.^19^ Park et al.^20^ reported that ethanol and its nonoxidative metabolites, not ACE, promoted acute alcohol‐induced liver, which was consistent with our present results. We found that there were no significantly difference of 4‐Hydroxynonenal among all experiment groups, which proved that alcohol of single intake was mostly metabolized through the non‐oxidative pathway.

It has been reported that the alcohol‐induced damage in liver significantly increases the production of cytokines, chemokines, other soluble mediators and components of the innate immune system,^17^, ^21^ which result in the alcohol‐stimulated liver fibrosis.^22^, ^23^ The K‐means cluster analysis of the present study indicated that the best clustering result of the DEPs was 2 clusters. The up‐regulated proteins of cluster 2 were involved in responses to stress, defence, activation of immune response and inflammatory response. Our results indicated that in the absence of AKR7A5, single ethanol binge can cause a more severe inflammatory response.

**FIGURE 6:**
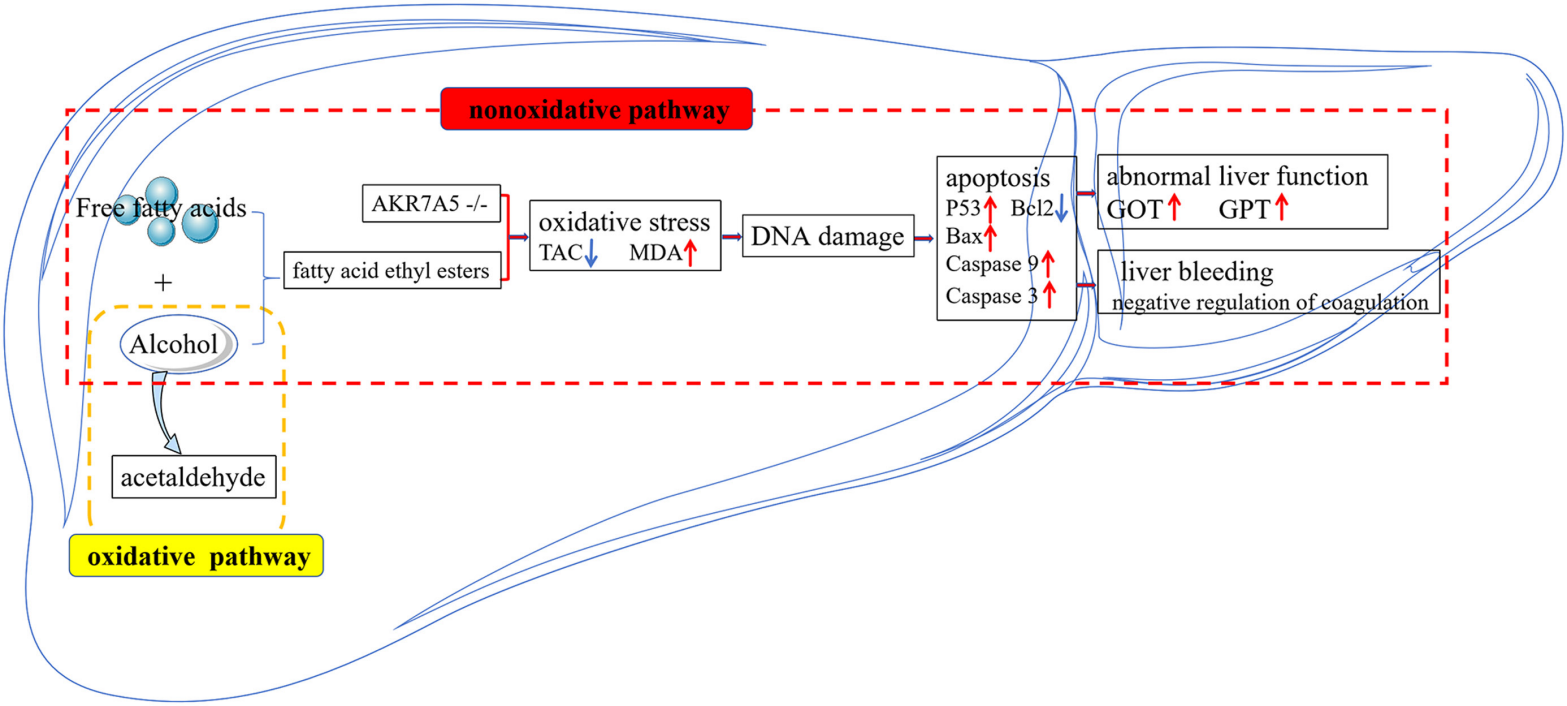
Cellular response in mice liver under the acute alcohol injury. The metabolic process of alcohol in the liver contains oxidative and non‐oxidative pathways. And single ethanol binge belonged to the non‐oxidative pathway, which induced acute liver injury (abnormal liver function and liver bleeding) through the occurrence by sequence of oxidative stress, DNA damage and apoptosis. GOT, Aspartate aminotransferase; GPT, Alanine aminotransferase; MDA, Malonaldehyde; TAC, Total antioxidant capacity.

Park et al.^20^ also proposed that single ethanol binge promoted acute alcohol‐induced liver injury by inducing endoplasmic reticulum oxidative stress, adipocyte death and lipolysis. So we detected the oxidative stress and apoptosis of livers from wild type and AKR7A5−/− mice under the alcohol treatment. And we speculated that oxidative stress and apoptosis would likely play a major role in the liver injury of AKR7A5−/− mice with a single ethanol binge. To investigate this, we monitored the antioxidant capacity (GSH, SOD, total antioxidant capacity), oxidation intermediates (MDA) and DNA damage of cells. The results demonstrated that under the alcohol treatment, the knockout of AKR7A5 caused the occurrence of oxidative stress, which induce the DNA damage. The damaged DNA up‐regulated the expression of P53, which induced the apoptosis of endogenous pathway (down‐regulated expression of Bcl2, up‐regulated expressions of Bax, Caspase 9 and Caspase 3) (Figure 6). In addition, we mined two interaction modules within the PPI network. And the BP enrichment analysis of module 1 was related to the metabolism of fatty acid, unsaturated fatty acid metabolism, long‐chain fatty acid metabolism, arachidonic acid metabolism, olefinic compound and icosanoid. The results of the present study indicated that the deletion of AKR7A5 caused more serious oxidative stress, apoptosis of endogenous pathway and abnormal lipids metabolism in mice liver under a single ethanol binge.

It was worth noting that there was a significant feature of congestion in the hepatic sinus and the space of hepatic cord in the liver of WT + alcohol and KO + alcohol groups compared with WT group. And the congestion particularly serious in the liver of KO + alcohol group. Coincidentally, two interaction modules were mined within the PPI network. And the BP enrichment analysis of module 2 was more enriched in the negative regulation of coagulation and haemostasis. The hub proteins of module 2 contained CRP, Proc, F2 and PF4. CRP, as a conserved host‐defence molecule, can inhibit the deleterious activities of phosphocholine‐ligands, such as modified low‐density lipoprotein and apoptotic cells and arrest the production of membrane‐damaging last product of the complement pathway.^24^ Proc plays a key role in haemostasis. The abnormal expression of Proc caused embryonic lethality or birth death from bleeding events.^25^ F2 is a vitamin K‐dependent proenzyme involved in the coagulation cascade. The deficiency of F2 is related to a rare, inherited bleeding disorder.^26^ PF4 is a highly/tetrameric protein, which can activate haemostasis through charge neutralization, and the dimeric binding of IgG molecules.^27^ In summary, the hub proteins (Proc, F2, PF4) found in the present study are reported to relate to the blood coagulation, which could well explain the reason of congestion in the hepatic sinus and the space of hepatic cord in the liver of KO + alcohol group. Our results prompted that the knockout of AKR7A5 caused the coagulation disorder.

## 5 CONCLUSIONS

This work provides substantial proteomic evidence that facilitates a deeper understanding of the impact of AKR7A5 on acute alcoholic liver injury, and identifies the major outcomes of AKR7A5 knockout under the single ethanol binge, including severe inflammatory response, oxidative stress, apoptosis of endogenous pathway, abnormal lipids metabolism and disordered coagulation in mice liver. It will facilitate for mechanistic understanding of alcohol‐liver disease and enrich the understanding of the function of AKR7A5.

## AUTHOR CONTRIBUTIONS

**Hui Shi:** Formal analysis (equal); investigation (equal); methodology (equal); project administration (equal); writing – original draft (lead). **Wenda Xu:** Investigation (equal); methodology (equal); project administration (equal); software (equal). **Qingling Liu:** Investigation (equal); methodology (equal); project administration (equal). **Yan Li:** Methodology (equal); writing – review and editing (equal). **Silin Dong:** Investigation (equal). **Zhenjun Zhao:** Investigation (equal); writing – review and editing (equal).

## CONFLICT OF INTEREST STATEMENT

All authors declare no competing interests.

## Data Availability

The data underlying this article are available in the article.
